# Neurological complications are avoidable during CABG

**DOI:** 10.12669/pjms.341.14114

**Published:** 2018

**Authors:** Zulfiqar Haider, Anjum Jalal, Asif Rashid Alamgir, Irfan Rasheed

**Affiliations:** 1 Zulfiqar Haider, FRCS. Department of Cardiac Surgery, Faisalabad Institute of Cardiology, Faisalabad, Pakistan; 2 Anjum Jalal FCPS-CS, FRCS-CTh. Department of Cardiac Surgery, Faisalabad Institute of Cardiology, Faisalabad, Pakistan; 3 Asif Rashid Alamgir, MS. Department of Anesthesia, Faisalabad Institute of Cardiology, Faisalabad, Pakistan; 4 Irfan Rasheed, MRCS. Department of Cardiac Surgery, Faisalabad Institute of Cardiology, Faisalabad, Pakistan

**Keywords:** Stroke, Coronary Artery Bypass, Atheroembolism, Off-pump, Aorta, Cardio-pulmonary bypass, Morbidity

## Abstract

**Objective:**

To review the incidence of stroke in patients undergoing CABG and the impact of a preventive strategy adopted at tertiary care unit of cardiac surgery.

**Methods:**

The data of all patients who underwent isolated CABG (N= 722) from July 2016 to August 2017 at Faisalabad Institute of Cardiology was retrieved for this retrospective study. All operations were done on cardiopulmonary bypass and cold blood cardioplegia. Numeric data was summarized as Mean ± Standard Deviation while categoric variables were summarized into frequency and percentage.

**Results:**

Mean age of patients was 53.83±8.8 years. Mean Parsonnet and Logistic EuroScore were 4.3±3.2 and 3.3±0.9 respectively. Forty nine patients (6.78%) had significant carotid artery disease. Mean number of grafts was 2.8±0.82. Diabetes was present in 27.8% patients. Neurological complications were noticed in 14 patients (1.94%) who included 12 permanent paralyses. Further subgroup analysis revealed that 67 patients who were operated by single clamp technique remained free of neurological complications. This is clinically remarkable finding but due to small population size it is statistically non- significant.

**Conclusion:**

The incidence of neurological complications can be reduced significantly by adopting the appropriate preventing measures. Use of Single Clamp technique may be the reasons of such a low incidence of stroke in this study.

## INTRODUCTION

The avoidance of neurological complications following coronary artery bypass grafting (CABG) is a major challenge in cardiac surgery. A multitude of factors is involved in the origin of these complications which makes its prevention extremely difficult.^1^ Majority of strokes occur intra-operatively and are largely attributed to the handling of aorta. The judicious use of anti-platelets, use of arterial filters in the cardiopulmonary bypass circuit and rigorous intra-operative hemodynamic management can help in minimizing neurological complications. Moreover, strategies to minimize the handling of aorta including Off-pump Coronary Artery Bypass (OPCAB), use of anastomotic devices and construction of both proximal and distal anastomoses on single clamp are thought to reduce the incidence of stroke. The single clamp technique has been reported to have potential to decrease the incidence of stroke but the evidence against this potential benefit has also been documented.^2^-^4^ In this study we have reviewed the outcome of our strategy for minimizing the neurological complications which is based on the evidence available in the literature.

## METHODS

This study is based on retrospective analysis of all patients who underwent coronary bypass grafting at Faisalabad Institute of Cardiology from July 2016 to August 2017. The data was retrieved from our dedicated cardiac surgery database (Cascade Cardiac Surgery 2005, Cascade Databases, Lahore) and exported to Excel spread sheet.

A total of 795 patients of CABG were found in the database. The records were further validated by physical search of patient's hospital files. A total of 722 patients were found to have isolated CABG and were included in the analysis. The remaining 73 patients had combined procedures and were therefore excluded from the study.

These operations were performed by four consultant cardiac surgeons. All patients had detailed preoperative work-up including full blood count, renal function tests, liver function tests, viral profile, echocardiography and abdominal ultrasound. The Cerebro-vascular accident (CVA) was defined as ‘neurological dysfunction affecting ambulation or day to day function'. Transient ischemic attack (TIA) was defined as any neurological deficit which disappeared completely within 24 hours.

The anesthetic management was done by same technique in all patients under direct supervision of one experienced consultant anesthetist (ARA). During the whole procedure, ECG, invasive blood pressure, central venous pressure and oxygen saturation were continuously monitored.

All patients underwent surgery with the help of cardiopulmonary bypass (CPB) that was established with an aortic cannula and a single venous cannula. The cardiopulmonary bypass circuits essentially used membrane oxygenators equipped with arterial filters. The systemic temperature was lowered to 32C and if required to 30C in cases where prolonged cross clamp was anticipated. Myocardial protection was achieved with ante-grade sanguineous cardioplegia solution using the Del-Nido solution. The Del Nido solution is mostly used in pediatric cardiac surgery but it was adopted by our group in adult cardiac surgery after carefully reviewing its safety profile in reported literature.^5^ Heparin was administered at a dose of 300 U/Kg and was repeated as required. One surgeon (AJ) in the study preferentially did CABG by using the technique of single clamp whenever possible. In this technique both proximal and distal ends were anastomosed with aortic cross clamp on. The rest of surgeons performed proximal anastomoses by applying partial occluding clamp separately for each graft (multiple-clamp technique).

The patients who developed neurological dysfunction after surgery underwent detailed neurological assessment and CT-Brain. The CT brain was repeated after 48 to 72 hours if the initial CT did not have any positive findings despite neurological deficit. Further evaluation and management plan was worked out with the help of visiting consultant neurologists.

Our strategy to avoid neurological complication essentially included the following:

1. All patients were kept on Aspirin till the day of surgery. The Clopidogrel was however stopped 4-days before operation in those patients who were on double anti-platelet therapy.
2. All patients received 150 mg Aspirin within 6-hours of operation.
3. Heparin was not reversed in patients who had coronary endarterectomy or were found to have diffuse disease with calcification of coronary arteries or carotid artery disease.
4. Arterial filters were used in the bypass circuits of all patients.
5. The mean blood pressure was strictly kept above 60 mm Hg while on bypass
6. The transfusions of whole blood or blood products were avoided whenever possible.

## RESULTS

The preoperative numeric variables of the study population are summarized in Table-I. The mean age is 53.83 years. The mean ejection fraction, serum creatinine and hemoglobin levels were all within normal range. The Parsonnet, Additive Euroscore and Logistic Euroscore were all below five which mean the patients had very low predicted mortality and hence belonged to low risk group. Only 49 patients showed some degree of carotid disease.

**Table-I:** Preoperative patient characteristics: Continuous variables.

Variable	N	Mean	Median	Stand Dev
Age	722	53.83	54.00	8.80
BMI	722	28.79	28.00	8.47
LVIDD	658	49.38	49.00	18.45
LVIDS	644	32.64	32.00	6.43
EF	711	52.70	60.00	10.17
Right Carotid -Stenosis	49	14.90	10.00	6.81
Left Carotid Stenosis	49	25.27	20.00	11.19
Creatinine	722	0.95	0.90	0.17
Haemoglobin	722	13.58	13.70	1.60
*Risk Score:*				
Parsonnet Score	722	4.30	3.00	3.02
Additive EuroScore	722	1.02	1.00	1.06
Logistic EuroScore	722	1.28	1.22	0.52

BMI: Body Mass Index, EF: Ejection Fraction, Hb: Haemoglobin.

The categorical variables are summarized in Table-II. It is obvious that the most of the patients presented with stable angina of CCS Class II-III. It was noted that 341 (47.16%) patients had experienced congestive cardiac failure in past while 4 (0.55%) patients were in cardiac failure at the time of surgery. Most of the patients (n=540, 74.69%) suffered from 3-vessel coronary artery disease while 19.36% (n=140) patients had 2-vessel disease and only 4.29% (n=31) patients had single vessel disease. In the study group very small number of patients i.e. 12 (1.66%) had significant left main stenosis? It was also observed that only nine patients (1.38%) had urgent surgery.

**Table-II:** Preoperative patient characteristics: Categoric variables.

Variable	N	%
*Gender*		
Male	595	82.30
Female	127	17.57
*CCS Class*		
Class I	69	9.54
Class II	524	72.48
Class III	121	16.74
Class IV	7	0.97
NA	2	0.28
*NYHA Class*		
Class I	228	31.54
Class II	402	55.60
Class III	87	12.03
Class IV	5	0.69
*Hypertension*		
Controlled	380	52.56
Uncontrolled	1	0.14
None	341	47.16
*Smoking*		
Still Smoking	1	0.14
Ex-Smoker	24	3.32
Non-Smoker	697	96.40
*Diabetes*		
Nil	522	72.20
Diet Controlled	1	0.14
On Tablets	13	1.80
On Insulin	186	25.73

CCS: Canadian Cardiac Society, ASA: American Society of Anesthetists, NYHA: New York Heart Association.

The unit follows a policy of aggressive risk factor modification before the CABG. It is therefore obvious that less than 1% patients were active smokers at the time of surgery. Similarly over 99% patients had normal blood pressure at the time of admission for surgery due to aggressive pre-operative medical management. Although the prevalence of diabetes was 28% in this study yet all had reasonable glycemic control before operation. These measures are known to reduce the risk of mortality which is obvious from the Risk Score calculated by the Parsonnet Score, Additive EuroScore and Logistic EuroScore (Table-I).

Operative & Post-operative characteristics are shown in Table-III. The mean bypass time is 104.54 minutes and cross clamp time is 65.28 minutes. These are within acceptable limits and ensure lower risk of pump related injuries. The vast majority of patients had three grafts as is evident by mean number of distal anastomosis which is 2.80. Almost all of the patients had at least one arterial graft utilizing LIMA, therefore, the mean and median number of proximal anastomosis are 1.92 and two respectively. The post-operative data are comparable with any internationally reputable center of cardiac surgery.

**Table-III:** Patient characteristics: Operative & post-operative.

Variables	N	Mean	Median	SD
*Operative*				
Bypass Time	722	104.59	98.00	42.55
Cross Clamp Time	722	65.28	62.00	34.83
Lowest Temperature	722	31.41	32.00	1.05
Proximal Anastomsis	722	1.92	2.00	0.87
Distal Distal Anastomosis	722	2.80	3.00	0.82
*Post-operative*				
ICU Stay (Hrs)	722	27.06	24.00	25.96
Ventilation Time (Hrs)	722	10.39	6.00	27.18
Inotrops (Hrs)	722	36.78	27.00	41.97
Chest Drainage (ml)	722	997.57	840.00	1707.52
Max CKMB	722	70.38	60.00	162.77
Hospital Stay (Days)	722	6.78	5.00	17.76
*Transfusions given*				
Whole Blood (units)	692	2.09	2.00	1.08
FFP (units)	343	1.22	1.00	0.79
Platelets (Units)	0	0	0	0.00

ICU: Intensive Care Unit, CKMB: Creatinine Kinase MB fraction, FFP: Fresh Frozen Plasma.

In this study only 14 patients (1.94%) developed any kind of neuro-psychiatric complications. These included 12 patients (1.66%) of permanent paralysis, one patient (0.14%) with transient ischemic attack and one patient (0.14%) with acute confusional state.

We conducted subgroups analysis of patients operated upon by one of the authors (AJ), who used two different techniques. This subgroup analysis involved a comparison between single and multiple clamp technique as shown in the Table-IV. The both subgroups were similar in their demographic profile (age, gender) and preoperative risk factors (ejection fraction, extent of coronary artery disease, renal profile, COPD) and operative variables namely (bypass time, Cross clamp time, number of grafts). Despite the homogeneity of patients, it is interesting to note that the patients who underwent surgery by single clamp technique (n=67) were clearly protected against such complications as none of them developed neurological complications. On the other hand the four out of 129 patients (4/129, 3.1%) operated by multiple clamp technique developed neuropsychiatric complications. There is clear clinical benefit of single clamp. However due to non random selection and relatively small number of patients the results have no statistical significance.

**Table-IV:** Single Vs Multiple Clamp Technique Single Surgeon (N=196).

Group	Neurological Complication	No Neurological Complications	Row Total
Single Clamp	0	67	67
Multiple Clamp	4	125	129
Column Total	4	192	196

P-value (0.30) by Fisher Exact test is not significant.

## DISCUSSION

The neurological complications of CABG have devastating impact on the life of patients as well as the cost of treatment. These complications are traditionally classified as Type-I and Type-II. Type-I complications result from a damage to the brain caused by embolic stroke, intracerebral bleed and present as paralysis, stupor or coma whereas the Type-II complications include decreased intellectual function or seizures.^6^ The incidence of neurological complications reported in literature varies significantly. Roach et al reported an incidence of 6.1% in their study of 2108 patients operated at 24 institutes of United States.^7^ The SYNTAX study has shown 2.2% incidence of early stroke in CABG vs. 0.6% in PCI.^8^ However, the incidence of type-II complications especially the cognitive dysfunction are probably much higher. Newman et al.^9^ have reported a post-operative incidence of cognitive decline up to 53% which gradually improves over next six months but persists in up to 24% of patients. Cognitive changes are very subtle and need expert evaluation. Nevertheless, during the counseling sessions by the cardiologists the patients find these figures so alarming that they tend to prefer percutaneous coronary intervention (PCI) over the CABG. This is one of the reasons that the drug eluting stents have resulted up to 20% decrease in the referral for CABG.^10^

Neurological injury during cardiopulmonary bypass has been attributed to several mechanisms including systemic inflammatory response, hypo-perfusion and micro embolism.^11^-^14^ For that particular reason the advent of Off-pump Coronary Artery Bypass (OPCAB) was associated with very high expectations of lowering the incidence of neurological complications. Unfortunately, the evidence has failed to prove this anticipated benefit of OPCAB.^15^-^17^

Handling of aorta during CABG is another factor considered to be the cause of neuro-cognitive defects. The application of cross clamp as well as the partial occluding clamp has been considered to cause micro-embolism from aorta and neurological dysfunction.^13^ If this is true, the total arterial OPCAB with a policy of no-touch to aorta should virtually eliminate neurological complication but there is little evidence in its support. Alternately, the on-pump beating heart surgery which eliminates the use of cross clamp should provide protection against neurological deficit as claimed by Sabban et al.^18^ In another retrospective study of 2,327 cases of coronary revascularization, Patel et al.^19^ have reported the prevalence of focal neurological deficit to be 1.6% in the on-pump group, 0.5% in the off-pump without aortic manipulation group while 0.4% in the off-pump with aortic manipulation group (p=0.027). Hence the evidence remains inconclusive and conflicting in this regard. The avoidance of partial occluding clamp is another strategy with theoretical benefits reported as early as 1982 by Heaton & Salerno^2^ and was supported again by Aranki et al. in 1994.^3^ However there have been fears of prolonged cross clamp time and its potential hazards of myocardial damage as reported by Kima et al. in 2001.^4^

The improvement of cardiopulmonary bypass and development of modern cardioplegia solutions have provided opportunity to complete both distal and proximal anastomoses without removing the cross clamp. There is still paucity of evidence to support this approach. It is therefore time to revisit these concepts and to conduct a randomized controlled trial for comparing the results of single and multiple clamp techniques. However, due to smaller incidence of this complication a statistically remarkable study would require a very large number of patients. Our group has worked out a plan for such a study and would initiate it once the ethical approval is granted.

## CONCLUSION

The incidence of neurological complications can be reduced significantly by adopting the appropriate preventing measures. A policy of continuing the use of pre-operative anti-platelets, stringent management of intra-operative blood pressure, use of arterial filters, administration of Aspirin within 6-hours of surgery and use of Single Clamp technique may be the reasons of such a low incidence of stroke in this study.

### Authors` Contribution

**ZH**: Concept, study design and editing.

**AJ**: Study design, data collection, analysis and write-up, final approval.

**AAR & IR**: Data collection, write-up, editing of relevant sections.
